# Inhibition of indole production increases the activity of quinolone antibiotics against *E. coli* persisters

**DOI:** 10.1038/s41598-020-68693-w

**Published:** 2020-07-16

**Authors:** Ashraf Zarkan, Marta Matuszewska, Stephen B. Trigg, Meng Zhang, Daaniyah Belgami, Cameron Croft, Junyan Liu, Sawssen El-Ouisi, Jack Greenhalgh, James S. Duboff, Taufiq Rahman, David K. Summers

**Affiliations:** 1 Department of Genetics, University of Cambridge, Cambridge, CB2 3EH UK; 2 Department of Veterinary Medicine, University of Cambridge, Cambridge, CB3 0ES UK; 3 Department of Biochemistry, University of Cambridge, Cambridge, CB2 1QW UK; 4 Henan Centre for Disease Control and Prevention, Zhengzhou, 450016 China; 5 The Oxford College of Engineering, VTU Visvesvaraya Technological University, Bangalore, 560068 India; 6 Wellcome Sanger Institute, Wellcome Genome Campus, Cambridge, CB10 1SA UK; 7 Faculty of Fundamental and Biomedical Sciences, Paris Descartes University, 75006 Paris, France; 8 Department of Pharmacology, University of Cambridge, Cambridge, CB2 1PD UK; 9 Medusa Pharmaceuticals Ltd, London, UK

**Keywords:** Antimicrobials, Bacteriology

## Abstract

Persisters are a sub-population of genetically sensitive bacteria that survive antibiotic treatment by entering a dormant state. The emergence of persisters from dormancy after antibiotic withdrawal leads to recurrent infection. Indole is an aromatic molecule with diverse signalling roles, including a role in persister formation. Here we demonstrate that indole stimulates the formation of *Escherichia coli* persisters against quinolone antibiotics which target the GyrA subunit of DNA gyrase. However, indole has no effect on the formation of *E. coli* persisters against an aminocoumarin, novobiocin, which targets the GyrB subunit of DNA gyrase. Two modes of indole signalling have been described: persistent and pulse. The latter refers to the brief but intense elevation of intracellular indole during stationary phase entry. We show that the stimulation of quinolone persisters is due to indole pulse, rather than persistent, signalling. In silico docking of indole on DNA gyrase predicts that indole docks perfectly to the ATP binding site of the GyrB subunit. We propose that the inhibition of indole production offers a potential route to enhance the activity of quinolones against *E. coli* persisters.

## Introduction

The decreasing effectiveness of antibiotic therapy represents an unprecedented, worldwide threat to human and animal health. The most widely publicised, and best understood, aspect of this problem is antibiotic resistance. This involves genetic change, through mutation or horizontal gene transfer. The target organism is rendered immune to the antibiotic by inactivation of the drug, alteration of its target or export from the cell^1^. A less well studied but increasingly important aspect of the anti-bacterial problem is the ability of small sub-populations (< 1%) of genetically sensitive bacteria to survive high concentrations of antibiotic by entering a dormant or partially-dormant state. These cells are known as antibiotic persisters^2–4^. When treated with a bactericidal concentration of an antibiotic, persisters display a temporary, non-heritable phenotype where they survive but do not replicate. When the antibiotic therapy is withdrawn, persisters revert to the growing state, giving rise to a population characterised by the same antibiotic sensitivity as the original population. In contrast, resistant cells continue to grow and divide in the presence of the antibiotic and the resistance phenotype is heritable^5–7^.

The mechanisms by which persister cells enter a dormant state are not well understood. It has been suggested that the activation of chromosome-encoded toxin-antitoxin systems is an important mechanism^8^ although this has recently been questioned^9^. Links have also been made to the stringent response and carbon source transitions^10^. A few reports^11,12^ have suggested a role for the signalling molecule indole and this seems plausible because indole has been known for several years to induce reversible *E. coli* dormancy^13^.

Indole is an aromatic signalling molecule produced by over 85 species of bacteria encompassing Gram-negatives, Gram-positives, pathogens and non-pathogens^14^. It is produced from tryptophan by the enzyme tryptophanase (TnaA)^15^. There exist two modes of indole action: persistent or pulse. In persistent signalling, indole is present in the culture for an extended period at a relatively low concentration (< 1 mM). In pulse signalling, intra-cellular indole reaches a high concentration (50 mM) for a short period (10–20 min) during stationary phase entry^16,17^.

In this study we examine the role of indole in the formation of persisters against antibiotics that target DNA gyrase, a bacterial type II topoisomerase. DNA gyrase is comprised of two subunits, gyrase A (GyrA) and gyrase B (GyrB), which form an A_2_B_2_ complex in the active enzyme^18^. The supercoiling activity of DNA gyrase involves the breakage and reunion of both polynucleotide strands, mediated by the GyrA subunits^19^. The GyrB subunits are responsible for the ATPase activity required for supercoiling^20^. One class of gyrase inhibitors, quinolone antibiotics, targets the GyrA subunit, stabilising double-strand breaks in the DNA^21,22^. A second class of inhibitors, aminocoumarins, targets the GyrB subunit and inhibits enzyme activity without stabilising double-strand breaks^20^.

It has been known for some time that the indole concentration reached during pulse signalling is sufficient to inhibit supercoiling by DNA gyrase in vitro^23^. Interestingly, the inhibition of DNA gyrase by indole occurs without the accumulation of double-strand breaks, thereby mimicking aminocoumarin antibiotics and suggesting a potential interaction between indole and the GyrB subunit^19^. Consistent with this, it was proposed from an in silico analysis that a derivative of indolinone, an indole analogue, may bind to the GyrB subunit of DNA gyrase^24^.

In the present study, we compared the proportion of persisters in wild-type and indole-negative (Δ*tnaA*; tryptophanase knockout) cultures of *E. coli* BW25113 after treatment with a range of antibiotics that target DNA gyrase. The absence of indole production decreased the number of persisters surviving quinolone treatment, with ciprofloxacin showing the most pronounced effect. Restoration of the wild-type level of ciprofloxacin persisters was achieved by an experimentally-applied indole pulse but not by supplementation with the lower indole concentrations associated with persistent signalling. In silico docking of indole on DNA gyrase predicts that indole docks at the ATP binding site of GyrB, suggesting that indole prevents the stabilisation of double-strand breaks and induces cellular quiescence by inhibiting gyrase activity.

## Results

### Indole stimulates the formation of *E. coli* persisters against quinolone antibiotics

**Figure 1:**
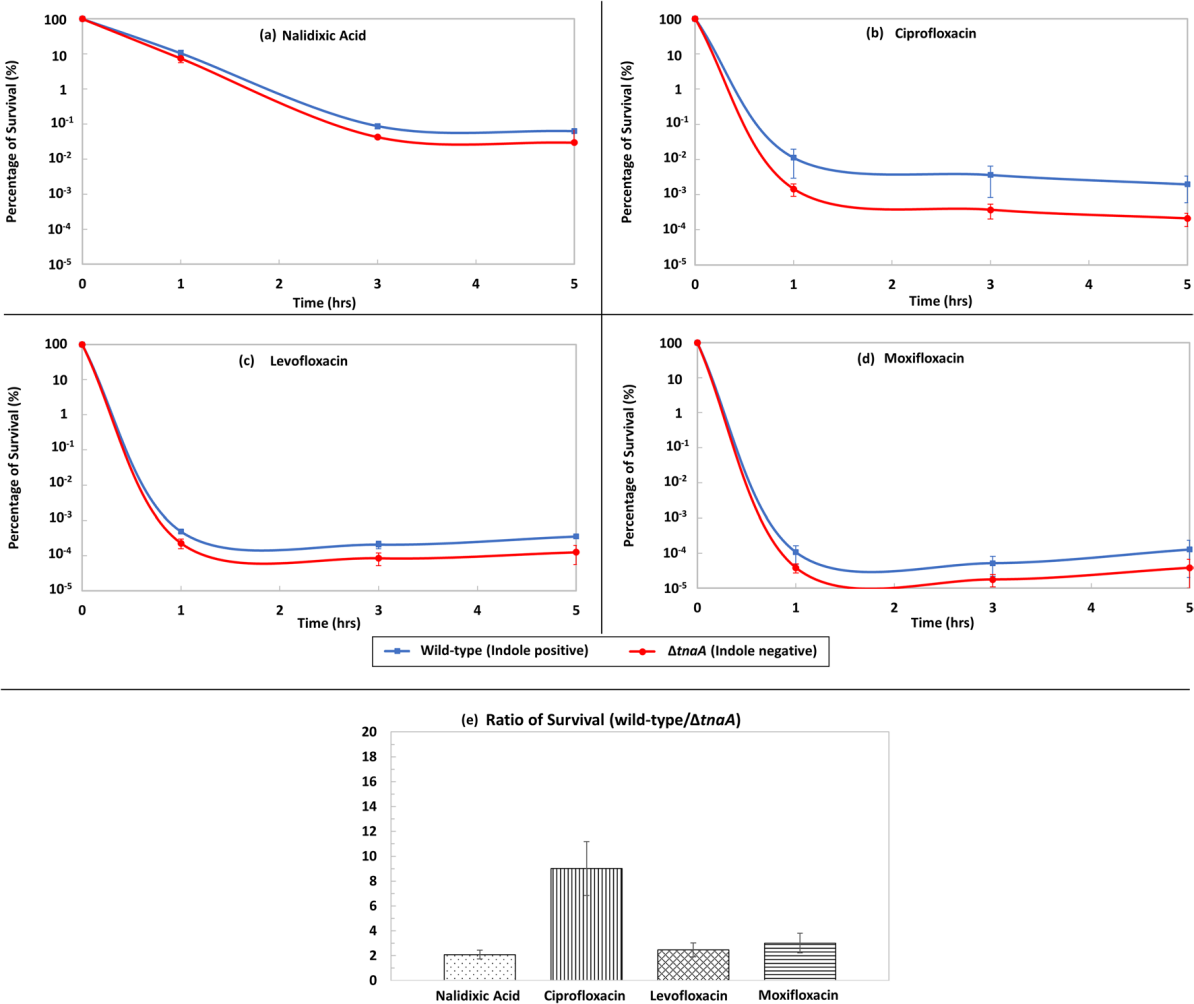
Indole enhances the formation of quinolone persisters in *E. coli*. Percentage of survival of exponential-phase (OD_600_ = 0.15) wild-type (indole positive) and Δ*tnaA* (indole negative) strains of BW25113 *E. coli* in LB medium treated for five hours with 100 × MIC (**a**) nalidixic acid, (**b**) ciprofloxacin, (**c**) levofloxacin and (**d**) moxifloxacin. The average ratio of survival (wild-type/Δ*tnaA*) in the plateau (3 to 5 h after antibiotic addition) for all four antibiotics is added for comparison (**e**). All data are the mean ± SE of a minimum of three biological replicates.

The frequency of persisters in wild-type and indole-negative (Δ*tnaA*) cultures of *E. coli* BW25113 was monitored after treatment with four quinolone antibiotics. One antibiotic was chosen from each of the four generations of quinolones: nalidixic acid (1st generation), ciprofloxacin (2nd generation), levofloxacin (3rd generation) and moxifloxacin (4th generation)^25,26^. The minimum inhibitory concentration (MIC) of each antibiotic was determined for both wild-type and Δ*tnaA* strains (Supplementary Information; Table S1). Antibiotics at 100 × MIC (sufficient to distinguish persistence from transient modes of resistance^5^) were added to exponentially growing *E. coli* cells for up to five hours and killing curves were generated. In each case there was a greater proportion of persisters in the wild-type cultures than the indole non-producing mutant. For nalidixic acid, levofloxacin and moxifloxacin the effect was relatively small (wild-type: Δ*tnaA* survival ratio approx. 2–4) with a bigger difference (approx. tenfold) effect for ciprofloxacin (Fig. 1). The result implies that in wild-type culture, 90% of ciprofloxacin persisters are indole-dependent. However, the fact that the indole-negative strain still produced persisters, confirms the existence of additional, indole-independent persistence mechanisms.

### Pulse signalling generates indole-dependent quinolone persisters in *E. coli*

If indole production stimulates the formation of persisters against quinolone antibiotics, it might be possible to restore the wild-type persister frequency in an indole-negative strain by adding indole to the culture. Indole exerts its effect through one of two signalling modes, persistent or pulse, and the effect of each of these was explored on an indole-negative strain.

**Figure 2:**
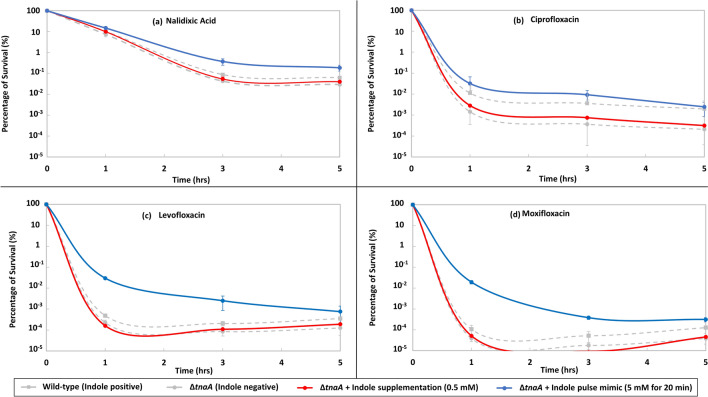
Indole pulse signalling stimulates quinolone persister formation in *E. coli*. Percentage of survival of exponential-phase (OD_600_ = 0.15) BW25113 Δ*tnaA E. coli* in LB medium treated for five hours with 100 × MIC **(a)** nalidixic acid, **(b)** ciprofloxacin, **(c)** levofloxacin and **(d)** moxifloxacin. For indole supplementation, 0.5 mM indole was added to *ΔtnaA* cultures prior to antibiotic addition. For the indole pulse, Δ*tnaA* cultures were treated for 20 min with 5 mM indole then washed and resuspended in fresh LB prior to antibiotic addition. All data are the means ± SD of a minimum of three biological replicates. Data for the percentage of survival of BW25113 wild-type and ∆*tnaA* in LB presented in Fig. 1 are included for comparison.

Persistent signalling occurs at indole concentrations found in stationary phase cultures. In LB cultures, where most indole is synthesised during stationary phase entry, this is in the range 0.3–0.7 mM^10,17,27–29^. To mimic this, LB cultures of the indole non-producing strain BW25113 ∆*tnaA* were supplemented with 0.5 mM indole. 100 × MIC of each of the four quinolone antibiotics was added and killing curves were generated over five hours of treatment. The killing curves for the ∆*tnaA*, with or without 0.5 mM indole, are not significantly different. The error bars of the curves for these two conditions clearly overlap (Fig. 2).

The effect of indole pulse signalling on the frequency of quinolone persisters was investigated by applying an artificial indole pulse. For wild-type cells growing in LB, the concentration of cell-associated indole during the pulse is around 60 mM^11^. An artificial indole pulse can be achieved by adding of 4–5 mM indole to the culture supernatant^16,17^ (Indole has a greater affinity for cells compared to the aqueous culture medium, which explains the difference in concentration between the cell-associated and the culture medium^16,17^). We treated exponentially growing cultures (OD_600_ = 0.15) of BW25113 ∆*tnaA* in LB with 5 mM indole for 20 min. The indole was removed by washing the cells in PBS before resuspending in fresh LB. 100 × MIC of each antibiotic was added and killing curves were generated over the next five hours (Fig. 2). Comparing ∆*tnaA* with and without the 5 mM pulse of indole, there was a significant difference, with no overlap of the error bars. The same result was obtained for all four antibiotics (Fig. 2). Another striking effect of the pulse was a reduction of the rate of cell death over the first three hours of antibiotic treatment; a phenomenon known as antibiotic tolerance^5^.

### The ionophore action of indole during the pulse is not responsible for the formation of quinolone persisters in *E. coli*

**Figure 3:**
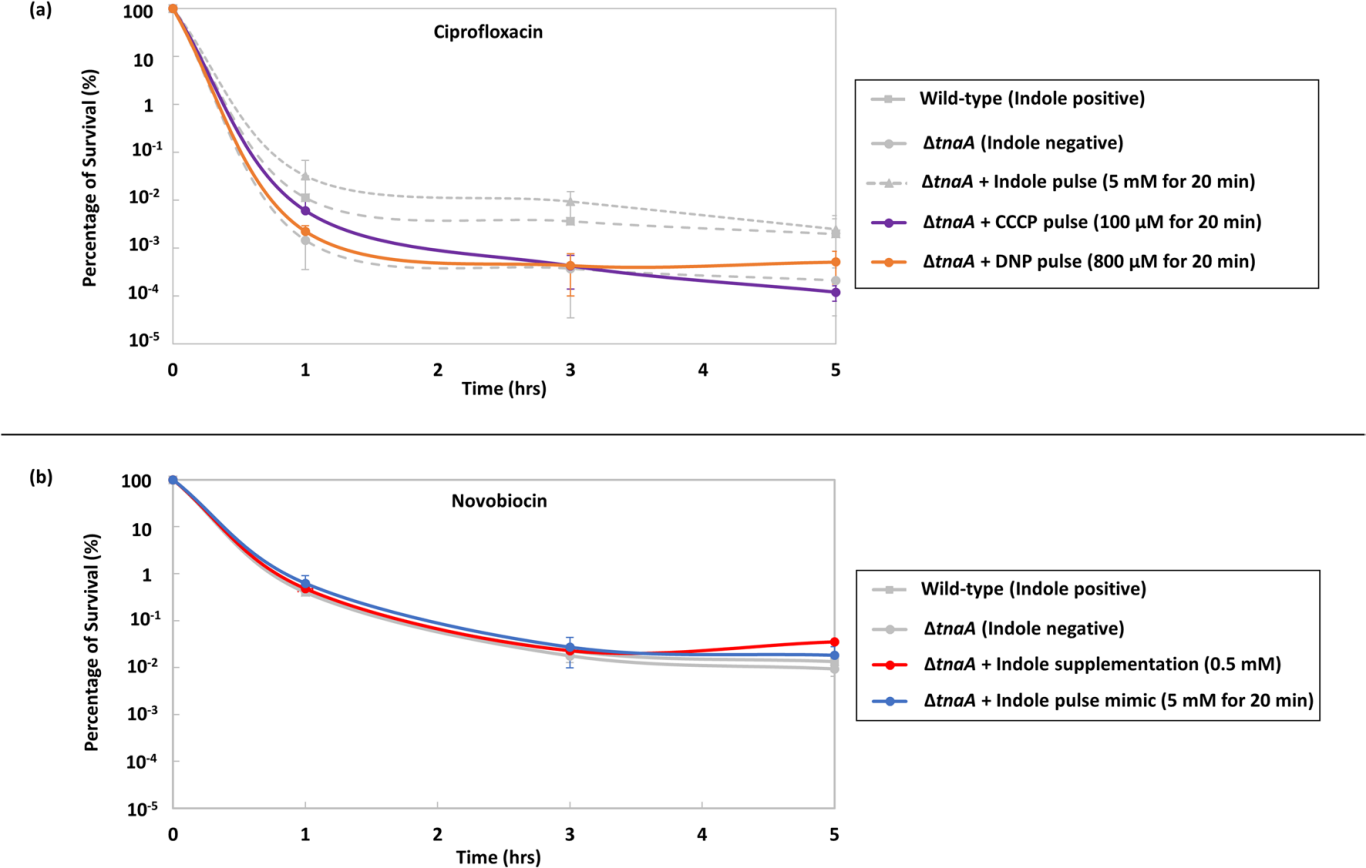
The frequency of *E. coli* persisters is not affected by (**a**) non-biological ionophores (CCCP and DNP) before ciprofloxacin treatment or (**b**) indole before novobiocin treatment. **(a)** Percentage survival of exponential-phase (OD_600_ = 0.15) BW25113 ∆*tnaA E. coli* in LB medium treated for 20 min with 100 µM CCCP or 800 µM DNP, then washed and resuspended in fresh LB and treated for five hours with 100 × MIC ciprofloxacin. Data for the percentage survival of BW25113 wild-type and ∆*tnaA*, with and without an indole pulse (Figs. 1 and 2), are included for comparison. All data are the mean ± SD of a minimum of three biological replicates. **(b)** Percentage survival of exponential-phase (OD_600_ = 0.15) wild-type (indole-positive) and Δ*tnaA* (indole-negative) strains of BW25113 *E. coli* in LB medium treated for five hours with 100 × MIC novobiocin. For indole supplementation, prior to antibiotic addition, Δ*tnaA* cultures were supplemented with 0.5 mM indole. To mimic an indole pulse prior to antibiotic addition, Δ*tnaA* cultures were treated for 20 min with 5 mM indole then washed and resuspended in fresh LB. All data are the mean ± SD of a minimum of three biological replicates.

At concentrations reached during pulse signalling, indole acts as a proton ionophore^13,16,29,30^. If membrane permeabilisation by indole is important for the formation of quinolone persisters, it should be possible to increase the frequency of persisters in an indole-negative strain by pulsing with an alternative ionophore. We therefore treated cultures of BW25113 ∆*tnaA,* growing exponentially in LB, with 100 µM carbonyl cyanide *m*-chlorophenyl hydrazone (CCCP) or 800 µM 2,4-Dinitrophenol (DNP) for 20 min. These chosen concentrations of CCCP and DNP have been previously shown to produces the same outcome as an artificial indole pulse^30^. The ionophore was then removed by washing the cells in PBS before resuspending in fresh LB. 100 × MIC of ciprofloxacin was added and killing curves were generated over five hours. Neither of the ionophores resulted in a significant increase in the frequency of persisters for the ∆*tnaA* mutant strain (Fig. 3a). Thus, we conclude that the ionophore action of indole is not sufficient for the formation of quinolone persisters in *E. coli*.

### Indole does not enhance *E. coli* persisters against an aminocoumarin antibiotic

We have shown that indole enhances the formation of quinolone persisters in *E. coli*. The active form of DNA gyrase is an A_2_B_2_ complex^18^ in which quinolones target the GyrA subunit^20–22^. In contrast, aminocoumarins, such as novobiocin, target the GyrB subunit of the enzyme^20^. To investigate whether indole also enhances formation of novobiocin persisters, 100 × MIC of the antibiotic was added to cultures of wild-type *E. coli* and ∆*tnaA* growing exponentially in LB and the killing curves were generated over five hours of treatment. Additionally, the effect of exogenous indole upon indole-negative cells was tested either by supplementing a culture with 0.5 mM indole or by applying a pulse of 5 mM indole for 20 min, following the procedures described earlier. Interestingly, there was no significant difference in the number of persisters after novobiocin treatment of wild-type and ∆*tnaA* strains*.* Moreover, neither indole supplementation (0.5 mM) nor an experimentally-applied pulse caused a significant change in the number of persisters with the ∆*tnaA* strain (Fig. 3b).

### Tryptophanase expression and indole production are not elevated in response to treatment with ciprofloxacin

**Figure 4:**
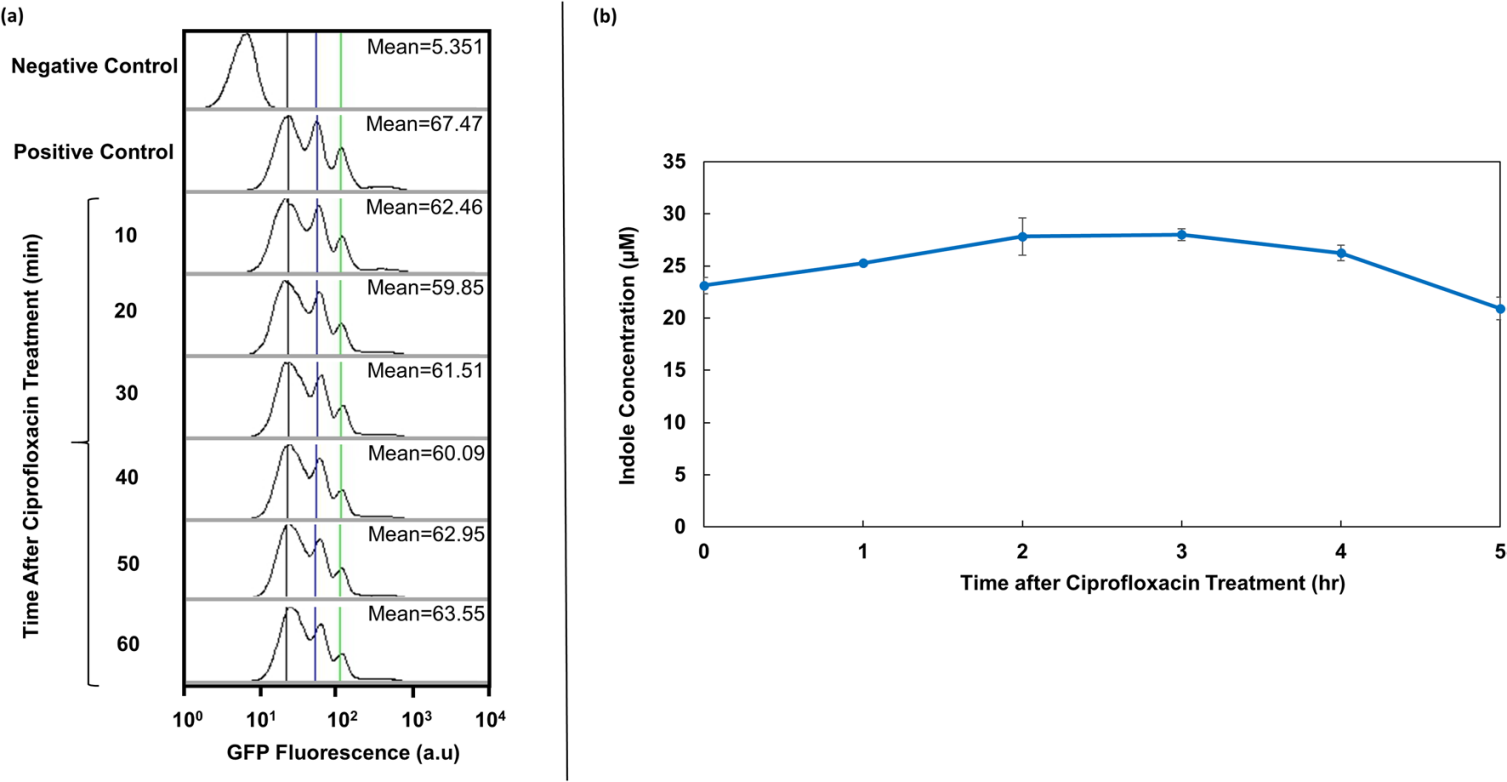
The expression of tryptophanase and the production of indole in *E. coli* are not affected by ciprofloxacin treatment. **(a)** Single cell distributions of tryptophanase-GFP. Exponential-phase samples (OD_600_ = 0.15) from LB cultures of *E. coli* BW25113 (negative control) and BW25113 TnaA-GFP were analysed by flow cytometry (100,000 events per sample). The fluorescence distributions of GFP in the TnaA-GFP culture is shown before (positive control) and after (10, 20, 30, 40, 50 and 60 min) the addition of ciprofloxacin (100 × MIC). In agreement with a previous study^31^, all samples showed 3 distinct peaks implying sub-populations of cells with low, medium and high tryptophanase content (peaks indicated by black, blue and green vertical lines, respectively). **(b)** Indole concentration (µM) in the supernatant of exponential-phase samples (OD_600_ = 0.15) from LB cultures of *E. coli* BW25113 wild-type before (time zero) and after (1, 2, 3, 4 and 5 h) treatment with ciprofloxacin (100 × MIC). Indole was measured by the Kovacs assay after passing each sample through a C18 solid phase extraction column to concentrate indole tenfold. All data are the mean ± SD of three biological replicates.

We tested whether indole production is increased in response to ciprofloxacin treatment. Increasing indole could be achieved either by increasing tryptophanase expression or by stimulating indole production by tryptophanase already present. We used an *E. coli* strain which expresses GFP-tagged tryptophanase from its native promoter^29^ to explore the effect of the antibiotic on enzyme expression. Ciprofloxacin (100 × MIC) was added to a culture growing exponentially and samples were taken over the next hour and analysed by flow cytometry. Both the mean fluorescence and the distribution of fluorescence among individual cells were similar to an untreated sample (Fig. 4a), indicating that ciprofloxacin treatment had no effect on the amount of tryptophanase in the cells.

Next, we investigated whether ciprofloxacin affects indole production. Indole concentrations are low (20–40 µM) in exponential phase cultures so a C18 solid phase extraction column was used as a pre-concentration step before performing the Kovacs assay^30^. Ciprofloxacin (100 × MIC) was added to an exponentially growing culture of wild-type *E. coli* and samples were taken over five hours of treatment for the measurement of supernatant indole. The concentration of indole in the supernatant before antibiotic addition was around 25 µM and showed little change during the treatment (Fig. 4b).

### Indole interacts with the GyrB subunits of DNA gyrase

Indole at 4–5 mM, when added to a bacterial culture, generates an intracellular concentration equivalent to that detected during pulse signalling. The same concentration inhibits DNA gyrase supercoiling activity in vitro without introducing double-strand breaks^19,23^. The indole moiety of an indolinone derivative has been shown to bind in the ATP-binding pocket of GyrB^24^, suggesting that indole might inhibit the ATPase activity of DNA gyrase. Gyrase inhibition, promoting dormancy without inducing DNA double-strand breaks, offers a plausible mechanism for the higher frequency of quinolone persisters seen in indole-producing bacteria and in non-producing strains subjected to an experimenter-applied indole pulse.

**Figure 5:**
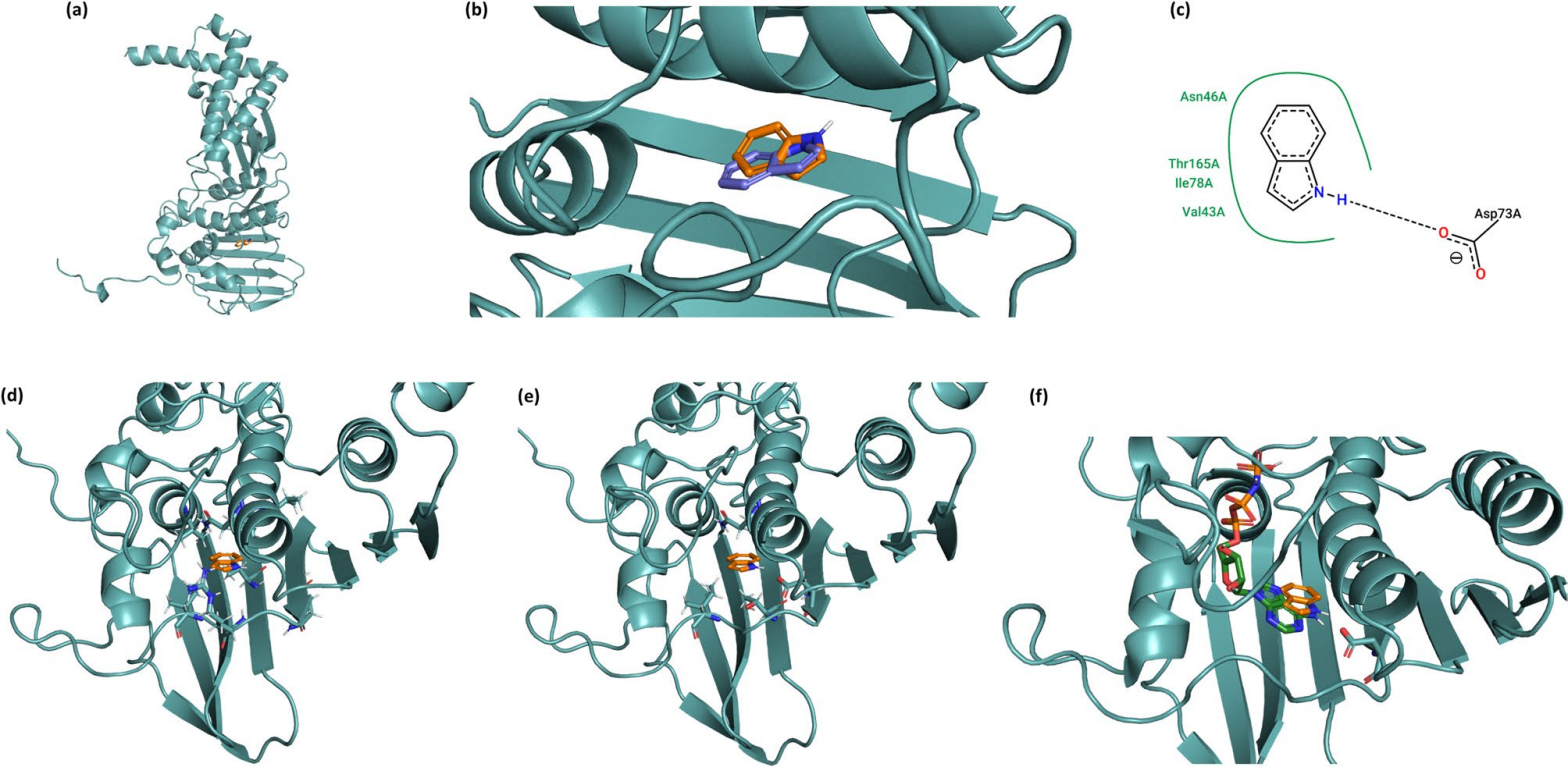
Molecular docking of indole in DNA gyrase predicts that indole docks at the ATP-binding pocket of GyrB. **(a)** The consensus docking of indole with DNA-gyrase B shown within structure 1EI1. **(b)** The consensus docked positioned of indole within 1EI1 (orange) and the consensus docked position of indole within 4PU9 (purple). **(c)** A 2D interaction diagram of the consensus pose in the binding pocket. **(d)** The consensus docked position of indole to DNA gyrase B within 1EI1, with residues identified by NMR^24^ shown in stick representation. **(e)** The consensus position of indole docked to DNA gyrase B shown within 1EI1, with key residues for the docked pose shown in stick representation. **(f)** The consensus docked positioned of indole within 1EI1 (orange), with aspartic acid D73 depicted in stick form. ATP analogue ADPNP (green) is shown in the 1EI1 structure. The close alignment between the indole and ADPNP nitrogen atoms responsible for hydrogen bonding to D73 is clearly visible. **(a–f)** were generated using PyMOL 2.2.2 (URL https://pymol.org/2/)^45^ while **(c)** was generated using PoseView (URL https://www.zbh.uni-hamburg.de/en/forschung/amd/server/poseview.html)^46^.

To investigate this potential indole-gyrase interaction, a preliminary docking exercise was performed using VIDA software (OpenEye Scientific Software) with the refined composite structure, P24, that was generated by Oblak and colleagues to dock indoline derivatives^24^. The modelling showed indole docking at the core of the ATP-binding site of GyrB. Following the preliminary exercise, docking in Autodock Vina was performed with structures with protein data bank (PDB) numbers 1EI1, 4WUD, and 4PRV to provide a strong consensus pose (Fig. 5a–c). 1EI1 and 4WUD gave docking within an RMSD of 0.7 Å of the consensus pose in more than 95% of repeats. Docking to 4PRV was less reproducible, however the most common docking site was consistent with the other structures (46% within an RMSD of 0.8 Å). 4PU9, which gave a consistent pose (98% within an RMSD of 1.5 Å), deviated from the others by a RMSD of 1.38 Å. Docking in LeDock supported the Vina consensus, with all structures yielding a result within a RMSD of 1 Å of the Vina consensus pose, with LeDock scoring an average of − 3.14 kcal mol^−1^ and the Vina consensus pose scoring − 5.8 kcal mol^−1^. The consensus pose corresponds well to indolinone derivatives docked previously, and is situated between the residues implicated in their binding by structural NMR^24^ (Fig. 5d,e). Further, the pose aligns the indole nitrogen responsible for hydrogen bonding to residue D73 with a nitrogen on adenine of ADPNP when bound to DNA gyrase subunit B, which also forms a hydrogen bond to D73 (Fig. 5f).

## Discussion

Our experiments demonstrate the existence of an indole-dependent mechanism for the formation of *E. coli* persisters that survive high-dose quinolone treatment. The indole-dependent effect is most pronounced for ciprofloxacin where persisters are reduced ten-fold in an indole-negative strain (Fig. 1).

The effect of indole on the formation of ciprofloxacin persisters seems to be entirely dependent upon the pulse signalling mode. Supplementation of the growth medium of an indole-negative (tryptophanase knock-out) culture with 0.5 mM indole (representative of indole in LB stationary phase culture) failed to restore the wild-type level of persisters (Fig. 2). However, an externally-applied indole pulse restored the number of ciprofloxacin persisters to the wild-type value (Fig. 2). Interestingly, the external pulse also increased persisters and reduced the rate of killing by nalidixic acid, levofloxacin and moxifloxacin.

We asked whether ciprofloxacin itself triggers the indole pulse that enhances persister formation. However, we found that neither the level of the indole-producing enzyme tryptophanase, nor the concentration of indole in the culture supernatant, was altered by ciprofloxacin (Fig. 4). In the absence of antibiotics, an indole pulse occurs during the transition of an LB culture of *E. coli* from exponential to stationary phase and it seems likely that this is responsible for creating persisters cells that survive ciprofloxacin treatment during subsequent exponential growth. Thus, although the pulse of indole is transient, its effect appears to be long-lasting; a paradox that we return to later in this discussion.

At concentrations similar to those seen during pulse signalling, indole functions as a proton ionophore^13,16,29,30^. However, our experiments suggest that membrane permeabilisation is not the mechanism of ciprofloxacin persister formation, since an externally-applied pulse of a non-biological ionophore (CCCP or DNP) did not produce the same outcome as an indole pulse (Fig. 3a). We therefore explored an alternative possibility that persisters might arise from indole inhibition of DNA gyrase.

The DNA supercoiling reaction of DNA gyrase involves the binding of a strand of DNA, the G (or gate) segment, at the interface of GyrA and GyrB^32^. A section of DNA then wraps around DNA gyrase, bringing a second segment, the T (or transported) segment, into the gate and positioning it above the G segment^33^. DNA gyrase then cleaves the G segment, forming a covalent bond between DNA and an active tyrosine residue in the enzyme. Upon binding of ATP, the T segment is passed through the broken G segment^34,35^. ATP hydrolysis and the release of ADP resets the DNA gate for another T segment to be captured^35^. This mechanism presents a number of opportunities for inhibition by antibacterial drugs and, potentially, indole. The transient, covalently-linked enzyme and DNA may be stabilised, or ATP binding or hydrolysis may be targeted to prevent the closure of the gate and thus the loss of T segment capture. Quinolones and aminocoumarins are classes of drugs that take advantage of these two mechanisms, respectively^20–22^.

Indole inhibits DNA gyrase supercoiling activity without the accumulation of double-strand breaks. This is similar to the effect of aminocoumarins, such as novobiocin^23^, that target GyrB ATPase by blocking access to the ATP-binding site^36^. In addition, an in silico study by Oblak and colleagues proposed that a derivative of indolinone, an indole analogue, binds to the GyrB subunit of DNA gyrase^24^. Building upon this indirect evidence, our in silico docking exercise provided a strong indication that indole docks at the ATP-binding pocket of GyrB (Fig. 5). If indole binding to GyrB inhibits gyrase activity by a mechanism similar to aminocoumarins (i.e. through inducing the closure of the GyrB gate, preventing T segment capture) then indole might be expected to provide protection against quinolones. This is because T segment capture is a prerequisite for cleavage of the G segment and subsequent formation of a covalent bond with a tyrosine residue at the interface of GyrA and GyrB. It is this bond that is irreversibly stabilised by quinolones, thus preventing DNA release. At the same time, reduced DNA gyrase activity would inhibit DNA replication and transcription, inducing dormancy and promoting the formation of persister cells. In support of this hypothesis, we note that, indole has no effect on novobiocin persister formation (Fig. 3b), as would be expected if indole and novobiocin compete the same binding site. It is plausible that indole is acting as a competitive inhibitor of ATP binding, thus preventing T segment capture, or preventing release of G segment through lack of a hydrolysable bond to provide energy to progress the mechanism. One could argue that indole might function similarly to the non-hydrolysable analogue of ATP, ADPNP, in that it may close the DNA gate and prevent DNA capture^34,35^.

Although DNA gyrase inhibition appears to be a plausible mechanism of indole-mediated persister generation, it remains to be explained how persisters formed by a pulse of indole during stationary phase entry can still exist in exponential phase many hours after the pulse. One possibility is that during the pulse the transient cell-associated indole concentration is sufficiently high to cause a local re-folding of GyrB that renders the topoisomerase inactive. In support of this, it has been reported that GyrB is structurally unique, with an unconventional fold^37^, and its misfolding reduces DNA gyrase activity^38^. The cell would be rendered dormant and immune to ciprofloxacin until the defective gyrase was either refolded (e.g. via chaperone action) or resynthesised. Given that indole derivatives such as compound 1 and indoline bind with affinities of 10 µM and 10 mM, respectively^24,39^, perhaps indole may bind with a similar affinity to the latter.

The structural differences between quinolones also present an interesting line of enquiry. The results of this study have shown that four quinolones generate persisters via an indole-dependent mechanism. However, the largest difference in the number of persisters between the indole producing and non-producing strains was seen with ciprofloxacin and, to a lesser degree, moxifloxacin (Fig. 1). This may be due to a major structural modification in both ciprofloxacin and moxifloxacin that is absent from nalidixic acid and levofloxacin, a cyclopropyl group at position 1 that is part of the enzyme–DNA binding complex and considered the most effective modification to this site^40^. Interestingly, a hydrogen or fused ring at position 8 has also been reported to potentiate quinolone targeting towards topisomerase IV, and moxifloxacin has a methoxy group at this position, which might increase targeting to DNA gyrase^40^.

In conclusion, our work has demonstrated the existence of an indole-dependent mechanism for the formation of quinolone persisters that is particularly effective for the fluoroquinolone ciprofloxacin. Evidence presented here suggests it involves the inhibition of DNA gyrase by high concentrations of indole that are seen during stationary phase entry. It is important to note that some persisters are seen, even in an indole-deficient (Δ*tnaA*) strain, which suggests that there is also an indole-independent mechanism of persister formation in operation. Nevertheless, the data suggest that the investigation of indole inhibitors as a method to reduce indole-dependent persisters in *E. coli* could have a major clinical relevance, for example in the treatment of urinary tract infections (UTIs) caused by uropathogenic *E. coli*. The effectiveness of indole inhibition in reducing *E. coli* persisters in UTIs could potentially be tested in vivo in the mouse model^41^ and could be particularly relevant in cases where *E. coli* persisters are linked to the relapse of infections following the withdrawal of treatment.

## Experimental procedures

### Chemicals and antibiotics

All chemicals and antibiotics were purchased from Sigma, except MIC Etests which were purchased from bioMérieux. Antibiotics were prepared as stock solutions in water, filter-sterilised, and stored as 1.5 mL aliquots at − 20 °C. A 1 × phosphate buffered saline (PBS) solution was used in all washing steps. Indole stock solutions (500 mM) were prepared in absolute ethanol and an ethanol control was included in all assays were the indole stock was used.

### Strains and culture conditions

All experiments were performed using *E. coli* K12 BW25113 wild-type, a corresponding kanamycin resistant tryptophanase knockout and a derivative expressing TnaA-GFP from its native promoter (Supplementary Information; Table S2). Luria–Bertani (LB) medium was used for all experiments (Supplementary Information; Table S3) and kanamycin (50 µg ml^−1^) was added for Δ*tnaA* cultures. Cells were cultured routinely at 37 °C, with shaking at 120 rpm, in LB medium. Cultures were streaked to single colonies on LB agar plates and incubated overnight at 37 °C to generate stock plates. An independent colony was picked from the stock plate to inoculate LB broth and incubated overnight at 37 °C in the shaking incubator. Overnight cultures were diluted to OD_600_ = 0.01 in a fresh LB and allowed to grow to OD_600_ = 0.15 before being used for subsequent assays.

Indole supplementation and indole, CCCP and DNP pulse-mimic were performed as described previously^30^. Briefly, for indole supplementation assays, indole (dissolved in ethanol) was added to a final concentration of 0.5 mM to a culture of BW25113 Δ*tnaA*. Ethanol alone was added to controls where appropriate. To impose an artificial pulse signal, indole (5 mM), CCCP (100 µM) or DNP (800 µM) (all dissolved in ethanol) was added to BW25113 Δ*tnaA* at OD_600_ = 0.15; ethanol alone was added to controls where appropriate. Cells were incubated for 20 min at 37 °C in the shaking incubator and then harvested by centrifugation at 2755 g for 10 min (Eppendorf 5,810 R centrifuge). The supernatant was removed, and the cells were resuspended in an equal volume of 1 × PBS (pH 7.0). Traces of Indole, CCCP or DNP were removed by centrifugation and removal of the supernatant. Cells were resuspended in an equal volume of fresh LB before being used for subsequent assays.

### Persister assays (100 × MIC antibiotic challenge)

A time zero sample was taken when cultures reached an OD_600_ of 0.15, immediately before the antibiotic (100 × MIC) was added. The flask was placed in a 37 °C shaking incubator for five hours and samples were taken after 1, 3, and 5 h of treatment and centrifuged for 7 min at 3,050 g at room temperature. After discarding the supernatant, the cell pellet was washed with an equal volume of 1 × PBS buffer to remove residual antibiotic and the pellet finally resuspended in an appropriate volume of PBS buffer. Washed samples were immediately serially diluted in 1 × PBS and 100 μl of two or more appropriate dilutions were spread on LB agar plates. This was carried out to limit the time bacteria were kept in the non-growing conditions after the antibiotic treatment. The plates were incubated in a static incubator at 37 °C and examined after 24 h to determine the cfu (colony forming units). The percentage survival was calculated by comparing the cfu of samples after 1, 3 and 5 h of treatment to the cfu at time zero.

### Flow cytometry

Samples were taken at OD_600_ = 0.15 before and after ciprofloxacin treatment. All samples were kept on ice for few seconds then analysed directly in a CyAn ADP (Beckman Coulter, Brea, CA, USA). GFP fluorescence was excited at 488 nm and measured through an emission filter of 530 ± 20 nm. For each sample, 100,000 events were collected at a rate between 2000 and 5,000 events per second. Data were collected using Summit 4.3 Software and analysed using R 1.0.136^42^ with the following packages: flowCore (data analysis), flowViz (plotting) and flowDensity (automated gating).

### Indole measurement by C18/Kovacs assays

Samples were taken at OD_600_ = 0.15 before and after ciprofloxacin treatment and centrifuged at 4 °C for 10 min at 3,050 g. The supernatant was removed, and the cell pellet was discarded. Indole concentration in the supernatant was measured using the C18/Kovacs method as previously described^30^.

Briefly, C18 solid phase extraction (SPE) cartridges (SampliQ C18, Agilent, CA, USA) were used to concentrate indole ten-fold before performing the Kovacs assay. Each cartridge (500 mg octadecylsilane – 6 ml) was equilibrated by flowing-through 10 ml 1-pentanol, followed by 10 ml deionised water. Each sample (50 ml culture supernatant) was flowed through an equilibrated cartridge and the cartridge was then washed with 10 ml deionised water. Indole was eluted from the cartridge with 5 ml 1-pentanol. 100 µl of × 10 indole concentrated samples (in 1-pentanol) were incubated with 150 µl of Kovacs reagent (10 g of *p*-dimethylamino-benzaldehyde dissolved in a mixture of 50 ml of HCl and 150 ml of amyl alcohol) for 5 min at room temperature. The reaction produced a soluble red product, which was measured spectrophotometrically at 530 nm (SpectraMax 190 Microplate reader; Molecular Devices, CA, USA). Eight known indole concentrations from 0 to 600 µM (in 1-pentanol) were assayed in triplicate and the mean results were used to construct a standard curve. Indole concentrations in unknown samples (also tested in triplicate) were calculated by comparison.

### In silico docking of indole in DNA gyrase

Indole was docked to DNA gyrase subunit B structures with the following PDB codes: 1EI1, 4PRV, 4PRX, 4PU9, and 4WUD with 100 replicates in Autodock Vina^43^. Further docking to the binding site identified in Autodock Vina was conducted in LeDock^44^, using the same structures. PyMOL^45^ was used for structure alignment, visualisation and figure generation, PoseView^46^ was used for 2D interaction diagram generation.

## Supplementary information

Supplementary file1 (PDF 184 kb)

## Data Availability

The data that support the findings of this study is available in Apollo (University of Cambridge Repository) at https://doi.org/10.17863/CAM.46130.
