# Supplementary material for: Inhibition of indole production increases the activity of quinolone antibiotics against E. coli persisters
Source: Sci Rep. 2020 Jul 16;10:11742. doi: 10.1038/s41598-020-68693-w (PMC7366635; doi:10.1038/s41598-020-68693-w)
Supplement: Supplementary file 1 — Supplementary file1 (PDF 184 kb) [file 41598_2020_68693_MOESM1_ESM.pdf]

SUPPLEMENTARY INFORMATION

Inhibition of Indole Production Increases the Activity of Quinolone Antibiotics against *E. coli* Persisters

Ashraf Zarkan[1,*,+], Marta Matuszewska[1,2,*], Stephen B. Trigg[1,3], Meng Zhang[1,4], Daaniyah Belgami[1,5], Cameron Croft[1], Junyan Liu[1,6], Sawssen El-Ouisi[1,7], Jack Greenhalgh[8], James S. Duboff[1,9], Taufiq Rahman[8] and David K. Summers[1]

[1]Department of Genetics, University of Cambridge, Cambridge, CB2 3EH, UK

[2]Department of Veterinary Medicine, University of Cambridge, Cambridge, CB3 0ES, UK

[3]Department of Biochemistry, University of Cambridge, Cambridge, CB2 1QW, UK

[4]Henan Centre for Disease Control and Prevention, Zhengzhou, 450016, China

[5]The Oxford College of Engineering, VTU Visvesvaraya Technological University, Bangalore, 560068, India

[6]Wellcome Sanger Institute, Wellcome Genome Campus, Cambridge, CB10 1SA, UK

[7]Faculty of Fundamental and Biomedical Sciences, Paris Descartes University, Paris, 75006, France

[8]Department of Pharmacology, University of Cambridge, Cambridge, CB2 1PD, UK

[9]Medusa Pharmaceuticals Ltd, London, UK

*These authors contributed equally and should be acknowledged as joint first author

[+]Correspondence: maa77@cam.ac.uk

Supplementary Methods

Minimum inhibitory concentration (MIC) assays

MIC assays for quinolone antibiotics were performed using Etest strips (BioMérieux, Marcy-l'Étoile, France) according to the EUCAST international guidelines (The European Committee on Antimicrobial Susceptibility Testing [EUCAST], 2014). Briefly, Mueller-Hinton agar plates (petri dishes containing 20 ml agar each) were seeded with an inoculum size equals to 0.5 McFarland standard (1.5 x 10^8 cfu/ml) of exponentially growing *E. coli* cells which corresponds to 1 ml of culture at an OD_{600} of 0.15. The plates were left to dry in a safety fume hood then an Etest strip was placed on the surface of each plate. The plates were incubated at 37 ºC and the results were obtained after 18 hrs incubation by taking the read at the line between the growth and the inhibitory halo.

MIC assays for novobiocin were performed using the broth microdilution technique according to the EUCAST international guidelines and ISO 20776-1 (2006). Briefly, 96 well plate containing a series of novobiocin concentrations in Mueller-Hinton broth were seeded with an inoculum size of 7.5 x 10^5 cfu/ml (in each well) of exponentially growing E. coli cells. The plate was incubated at 37 ºC with shaking at 120 rpm and the results were obtained after 18 hrs incubation by taking the read at the lowest antibiotic concentration where no growth was visually observed.

Supplementary Table. S1: Minimun inhibitory concentration (MIC) of antibiotics used in this study

Antibiotic	MIC (µg/ml)	
	**BW25113 wild-type**	**BW25113 Δ*tnaA***
Nalidixic Acid	4	4
Ciprofloxacin	0.012	0.012
Levofloxacin	0.094	0.094
Moxifloxacin	0.125	0.125
Novobiocin	8	8

Supplementary Table. S2: Bacterial strains used in this study

**Strain**	**Genotype**	**Reference**
E. coli K12 BW25113	*rrnB3 ΔlacZ4787 hsdR514 ΔaraBAD567 ΔrhaBAD568 rph-1*	[1]
E. coli K12 BW25113 Δ*tnaA*: Km^R	BW25113 Δ*tnaA* Ω Km^R	[1]
E. coli K12 BW25113 TnaA-GFP	*tnaA::gfp*; BW25113 tryptophanase tagged with GFP	[2]

Supplementary Table. S3: Luria-Bertani (LB) media used in this study

**Media**	**Component**	**Concentration (g/L)**
LB broth (Miller Formedium™)	NaCl<br>Yeast extract<br>Tryptone	0.5<br>5<br>10
LB agar (Miller Formedium™)	NaCl<br>Yeast extract<br>Tryptone<br>Agar	0.5<br>5<br>10<br>15

Supplementary References

1. Baba, T. *et al*. Construction of *Escherichia coli* K-12 in-frame, single-gene knockout mutants: the Keio collection. *Mol Sys Biol.* **2**, 2006.0008 (2006).
2. Gaimster, H. & Summers, D.K. Regulation of Indole Signalling during the Transition of *E. coli* from Exponential to Stationary Phase. *PLoS One*. **10**, e0136691 (2015).
